# Reducing Typhoid Burden within a Generation

**DOI:** 10.4269/ajtmh.18-0500

**Published:** 2018-07-25

**Authors:** Zulfiqar A. Bhutta, Anita K. M. Zaidi, Tikki Pangestu

**Affiliations:** 1 Centre for Global Child Health, The Hospital for Sick Children, Toronto, Canada;; 2 Dalla Lana School of Public Health, University of Toronto, Toronto, Canada;; 3 Center of Excellence in Women and Child Health, The Aga Khan University, Karachi, Pakistan;; 4 Vaccine Development and Surveillance, Bill & Melinda Gates Foundation, Seattle, Washigton;; 5 Enteric and Diarrheal Diseases, Bill & Melinda Gates Foundation, Seattle, Washigton;; 6 Visiting Professor, Lee Kuan Yew School of Public Policy, National University of Singapore, Singapore, Singapore

For us, the authors of this viewpoint, our respective journeys related to typhoid fever span almost three decades, from an early exposure to the severe morbidity associated with drug-resistant typhoid^1,2^ to assessment of priorities for dealing with the burden of a neglected disorder.^3^ Despite the fact that there have been numerous, mostly modeled, global estimates of the burden of typhoid over the past several decades,^4–6^ progress in addressing this illness has been woefully slow. This is partly a result of limited nationally representative estimates from low- and middle-income countries and general misperceptions that typhoid is largely preventable through socioeconomic development and gains in environmental conditions, such as sanitation and safe water, and that secular trends suggest that the disease burden is decreasing.

A range of recent investments have aimed to improve our collective knowledge of the real burden of typhoid in endemic areas. These include the landmark studies conducted in Asia under the International Vaccine Institute’s Diseases of the Most Impoverished (DOMI) program. Although focused on evaluating the feasibility of introducing the Vi polysaccharide vaccine, DOMI provided population-based estimates from various endemic countries in Asia.^7^ Although there is an agreement on progress and reductions in typhoid burdens in parts of Latin America and Southeast Asia over the last several decades, it is also notable that typhoid remains a significant disorder in South Asia^8^ and remains largely underestimated in sub-Saharan Africa.^9^ Also, most of the data sets used in global burden estimates to date have largely relied on their models on population-based estimates from a limited number of countries and from special studies.

Recent gains in our collective knowledge of typhoid burden have been supported to a large extent by investments from the Bill & Melinda Gates Foundation in ground breaking studies of the current typhoid burden, disease severity, and complications related to typhoid in Asia and Africa. Although there are relatively few nationally representative population-based estimates, these recent studies have been reliant on methodologies from reliable laboratories and research institutions aiming to provide robust estimates of blood culture proven typhoid. The current exercise summarized in the supplement included in this issue aimed to collect and synthesize information on blood culture–proven typhoid from a range of referral hospitals and laboratories to assess recent trends and correlates of typhoid. Participating country teams evaluated information on various social determinants and ecological trends, and assessed if there were clear trends in the burden of culture proven typhoid in the centers or populations evaluated.^10^

Despite several limitations of the country case studies, which included relatively few nationally representative estimates, several common findings emerged. In many instances, although the typhoid burden has reduced in comparison with the situation two decades ago (variable rates from annual reduction of 25% to an increase of 21%), rates seem to have stabilized to low levels in many countries. However, especially among South Asian countries, the burden is still substantial, accounting for 1–7% of all positive-blood cultures in South Asia from the laboratories sampled.^10^ The observed trends of typhoid also do not bear a clear relationship to economic development, education, or changes in coverage of water and sanitation interventions. Despite many common risks, no relationship was observed with diarrheal disease burden or trends. An important finding from the global review and case studies was the emergence and persistence of antimicrobial resistance (AMR) among typhoid isolates in several geographies, posing a challenge for access to appropriate therapy.

One would have expected that recent findings on typhoid would be commensurate with measures in countries or globally to address these issues. Instead, there appears to have been much inaction and lack of a clear global policy to address typhoid. Typhoid was largely ignored during the period of the millennium development goals, given the singular focus on maternal and child health; rather, it was orphaned within global organizations such as World Health Organization (WHO) and United Nations International Children’s Fund. Much of the typhoid-relevant work was relegated to the vaccines and biologicals program at the WHO, and despite the fact that the disease burden was disproportionately high among children, including children under 5 years,^11,12^ none of the programs within the WHO Child and Adolescent Health Department targeted appropriate diagnosis and management of typhoid.

Several elements need to be considered in charting a way forward for typhoid control. Notwithstanding the lack of clear evidence that economic and social development lead to reduction in disease burden in the short term, countries should continue to invest in improved sanitation services and safe water as key interventions. For much of South Asia this will mean addressing the continuing problems of open defecation, safe water access, and sewage disposal. Given population transition and demographic changes and migration patterns, addressing the issues of urban health, especially among slum dwellers, should be prioritized.

The emergence of AMR among strains of typhoid resistant to both fluoroquinolones and third generation cephalosporins^13^ poses special risks that must be addressed. This requires the development of protocols and appropriate diagnostics for rapid detection of typhoid, especially multidrug resistant typhoid among febrile cases in endemic areas (where common differential diagnoses include malaria and viral infections such as dengue or chikungunya), as well as financial support strategies to make this possible. The importance of mainstreaming typhoid diagnosis and treatment within routine health systems has been underscored in previous evaluations of health-care seeking patterns.^14,15^ Given the current focus on universal health coverage and mechanisms to provide safety nets to at-risk populations, this will require development of strategies for mainstreaming the management of typhoid fever, especially complicated cases requiring referral. Antimicrobial resistance continues to need global monitoring and, importantly, sharing of information between countries. More recent super-resistant strains of typhoid^12,13^ pose special problems, with higher morbidity rates and significantly higher cost of therapy compared with drug-sensitive infections. Managing such infections should go hand in hand with sound monitoring and surveillance systems to guide public policy and general practice in settings where obtaining blood cultures in all febrile cases is not possible.

There is now much more hope that a concerted and comprehensive strategy for typhoid prevention and control may be possible. These include the recent development of effective typhoid conjugate vaccines,^16,17^ recommendation by the WHO Strategic Advisory Group of Experts on Immunization for the use of newer typhoid conjugate vaccines,^18^ and the opening of a window for financing such initiatives by the Global Vaccine Alliance. There is now a clear pathway for the appropriate targeted use of the vaccine and its inclusion within the expanded program of immunizations for infants in endemic areas and in campaigns targeting at risk populations.

Given the known risks of typhoid in conflict settings^19^ and among displaced populations,^20^ appropriate immunization strategies also include the use of typhoid vaccines in humanitarian settings as part of a comprehensive response strategy. Given past experience with the use of typhoid vaccination in outbreak situations,^21^ this could be a feasible response. The recent successful use of a single-dose cholera vaccine in response to an outbreak in Zambia^22^ also offers a real opportunity for the use of typhoid conjugate vaccine in such settings.

In line with emphasis within the sustainable development goals on preventive strategies and universal health coverage, it is time to adopt a new action plan for the prevention and control of typhoid fever. This will require substantial focus on advocacy and action in countries at the highest risk, with updated information on disease burden, appreciation of specific contextual factors, which are amenable to public health investments, and preventive strategies including the newer vaccines. Not surprisingly, as underscored by the country case studies in this supplement, there was little awareness in countries of the preventive potential of vaccines,^10^ a status that the recent position statement from WHO^18^ might change. Developing and implementing a comprehensive global action plan for typhoid encompassing a range of preventive strategies and immunization approaches, and appropriate diagnosis and treatment strategies is an essential step in controlling typhoid within a generation.
